# Neurosurgical site infections: a retrospective monocentric study of pediatric brain tumor patients

**DOI:** 10.1007/s00381-025-06765-w

**Published:** 2025-02-24

**Authors:** Giorgia Enrico, Eleonora Fusco, Matteo Palmetti, Federico Mussa, Iacopo Sardi, Elena Chiappini, Luisa Galli, Elisabetta Venturini

**Affiliations:** 1 Pediatric Neuro-Oncology Unit, Meyer Children’s Hospital IRCCS, Florence, Italy; 2 Department of Health Sciences, University of Florence, Florence, Italy; 3 Department of Health Sciences, Postgraduate School of Pediatrics, University of Florence, Florence, Italy; 4 Department of Neurosurgery, Meyer Children’s Hospital IRCCS, Florence, Italy; 5 Paediatric Infectious Diseases Unit, Meyer Children’s Hospital IRCCS, Florence, Italy

**Keywords:** Neurooncology, Infectious disease, *Staphylococcus aureus*, Antibiotic therapy

## Abstract

**Purpose:**

This retrospective monocentric study aims to describe the characteristics of neurosurgical site infections (n-SSI) in neurooncological pediatric patients. The primary goal was to assess infection rates and identify common pathogens in this population.

**Methods:**

We considered pediatric patients (0–18 years) who underwent neurosurgery for brain tumors at Meyer Children’s Hospital in Florence between January 1, 2017, and December 31, 2021. Children with suspected/confirmed n-SSI were included. Data were retrospectively collected from patients’ medical records, and covered tumor and surgery type, presence of foreign bodies, microbiological findings, and treatment. Infections were classified into categories according to literature. Statistical analysis was performed using GraphPad Prism. A *p* value of < 0.05 was considered significant.

**Results:**

Of 352 children undergoing neurosurgery, 43 (12.22%) had suspected/confirmed n-SSI, with a confirmed infection rate of 4%. The most frequent n-SSI was postoperative meningitis (37.2%), followed by wound infections (25.6%). A foreign body was present in 74.4% of cases. The most prevalent pathogens were Staphylococcus aureus (40%) and coagulase-negative staphylococci (33%). Lumbar puncture (LP) performed before antibiotics significantly increased pathogen isolation (*p* = 0.01). Most patients (95.3%) had fever, and 53.5% had CSF leakage. Antibiotic therapy was administered in all cases, and 65.1% required therapy adjustment. No significant correlation was found between foreign body and clinical symptoms or microbiological positivity.

**Conclusion:**

The study reveals a high rate of n-SSI, emphasizing the importance of early diagnostic measures like LP to improve microbiological diagnosis and optimize antimicrobial treatment. The most frequent pathogen was *S. aureus*; however, the absence of methicillin-resistant strains is notable. These findings highlight the role of a multidisciplinary approach in managing n-SSI and the potential for n-SSI to delay adjuvant cancer treatments.

## Introduction

Surgical site infection is a relatively common complication of surgery; particularly, infections after neurosurgical procedures carry a high morbidity rate and may have a life-threatening potential [1]. Incidence, characteristics, and risk factors of neurosurgical site infection (n-SSI) in adult patients are very well described in scientific literature, but in the pediatric population such complication is currently not well defined [1–12]. Available pediatric studies agree that n-SSI is one of the main reasons for hospital readmission after brain tumor resection, especially in the first 30 days after surgery [13]. One of the main outlined risk factors in these studies is the presence of a foreign body implant, either synthetic dural or bone substitute, or a cerebrospinal fluid (CSF) shunt device [13–17]. The incidence of n-SSI associated with CSF shunt is about 11%, as reported in two pediatric cohorts. Premature birth, age 6–12 months, presence of gastrostomy tube, CSF shunt revision, previous shunt infection, and intraoperative use of the neuro-endoscope were all identified as independent risk factors at multivariate analysis [17, 18]. Moreover, patients with brain tumors seem to face an increased risk of infection and wound healing disturbances due to prolonged treatment with steroids and to the immunosuppressive effects of chemotherapy and radiation. The microorganisms most frequently associated with n-SSI are Gram-positive cocci, in particular *S. aureus* in 50–70% of cases [2, 3, 7] with methicillin-resistant *S. aureus* (MRSA) found in up to 75–80% of those cases [10, 15], and *coagulase-negative staphylococci* (CoNS), reported in 25–38% [1, 5, 8, 19]. CoNS meningitis is usually related to trauma or direct implantation of foreign bodies, or to the presence of a CSF shunt [20]. Differentiating between clinically relevant infections and possible contaminations could be challenging, as multiple cultures are required to confirm the infection [21].

To date, the use of antimicrobial agents for n-SSI prevention in pediatric patients is not standardized. Antimicrobial prophylaxis in adult and pediatric patients undergoing neurosurgery is currently recommended by scientific societies [10, 22], although a few studies showed that antibiotic prophylaxis did not affect meningitis incidence rate [6, 7].

The purpose of this retrospective study was to describe the epidemiological, clinical, microbiological, and therapeutic characteristics of children with brain tumors, seen at a referral tertiary care pediatric center, who presented a suspected/confirmed n-SSI.

## Material and methods

### Patient selection

A 5-year observational retrospective study has been conducted at the Neuro-oncology Unit of Meyer Children’s Hospital in Florence, one of the main pediatric hospitals and referral center for pediatric brain cancer in Italy. Children with brain tumors admitted to this hospital for suspected/confirmed n-SSI between 1 January 2017 and 31 December 2021 were considered eligible. Relevant information was entered into the study database in accordance with international standards for data protection. Data were retrospectively collected from patients’ medical records, and covered tumor type, surgical details, presence of foreign bodies, microbiological findings, and treatment; in particular, synthetic dural or bone substitutes and CSF shunt devices were considered foreign bodies. To reduce the recall bias, we excluded patients with insufficient information on medical records. This study was approved by the Meyer Children’s Hospital Pediatric Ethical Committee (ethics approval number 39/2024). All the parents of children gave their consent for inclusion in this observational study.

The National Healthcare Safety Network criteria for healthcare-associated infections were used to define healthcare-associated n-SSI if occurring within 30 days after surgery, or within 1 year if a synthetic implant was in place and the infection appeared to be related to the surgery procedure [10, 17]. In this study, n-SSI was defined according to the Infectious Diseases Society of America (IDSA) [23] and Centers for Disease Control and Prevention (CDC) [24] criteria.

### Definition and classification of n-SSI

To better standardize the results, n-SSI were classified into categories according to literature [5, 6, 10, 11, 23–25]. We also distinguished between confirmed and suspected infection for each of the following categories.

#### Wound infection (superficial or deep incisional)

We considered n-SSI a wound infection if any of the following signs were present: pain or tenderness, localized swelling, CSF leak, hyperemia, or heat, with or without fever (considered as suspected infection) or presence of an abscess or other evidence of infection involving the deep incision found on direct examination and/or on microbiological findings (considered as confirmed infection).

#### Postoperative meningitis

Postoperative meningitis was defined by either at least one CSF Gram stain, culture or *polymerase chain reaction* (PCR) demonstrating a microorganism (considered as confirmed infection); or presence of fever and/or headache plus meningeal signs and/or cranial nerve signs, associated with CSF leukocytosis, increased protein and decreased glucose concentration, or associated with organisms identified from blood (considered as suspected infection).

#### Postoperative brain abscess

Postoperative brain abscess was defined by a microorganism isolated from brain tissue or subdural space aspiration (considered as confirmed infection), or a surgical diagnosis of brain abscess, or fever and altered mental status and/or focal neurological deficit and suggestive computed tomographic scan (considered as suspected infection).

#### CSF device infection

A CSF device infection was considered in the case of isolation of a bacterial pathogen from an implanted shunting device after removal and any shunt malfunction symptoms, meningism or fever, or a positive culture on CSF analysis obtained through the device or by LP (considered as confirmed infection). LP was performed in all patients with suspected n-SSI infection involving CNS (e.g., meningitis, CSF shunt infection, dural substitute infection). Meningitis and general sepsis in patients with a device were counted as suspected shunt infections.

#### Dural substitute infection

A dural substitute infection was considered in case of isolation of a bacterial pathogen from the artificial cranial substitute after removal or from CSF, and signs of meningism and/or fever/CSF leak (considered as confirmed infection). Patients without microbial isolation who only removed the substitute because of clinical suspicion by the neurosurgeon were considered as “suspected” dural substitute infection. All the dural substitutes in our analysis were Gore-Tex DM® (W.L. Gore & Associates Inc., Phoenix, AZ, USA).

#### Bone-flap infection or osteomyelitis

This subgroup of n-SSI was defined as isolation of a bacterial pathogen from the free bone-flap placed in supratentorial craniotomy after its removal, or from bone, or evidence of osteomyelitis on direct examination of the bone during a surgical operation or histopathologic examination (considered as confirmed bone-flap infection), or presence of any signs (fever, localized swelling, tenderness, heat, or drainage at suspected site of bone infection) and radiographic evidence of infection and/or positive blood culture (considered as suspected bone-flap infection).

According to CDC, CoNS infections were confirmed in case of positivity of two different samples or in presence of clinical and/or laboratoristic signs of infection and microbiological identification in one sample. Categorical data were reported as absolute values and percentages and compared with the *χ*^*2*^ test or Fisher’s exact test. Continuous variables were reported as medians and interquartile ranges (IQR) and compared with the non-parametric Mann–Whitney test. A *p* value < 0.05 was considered statistically significant. Statistical analyses were performed using GraphPad Prism (Version 8.4.2, GraphPad Software, San Diego, CA, USA).

## Results

**Fig. 1:**
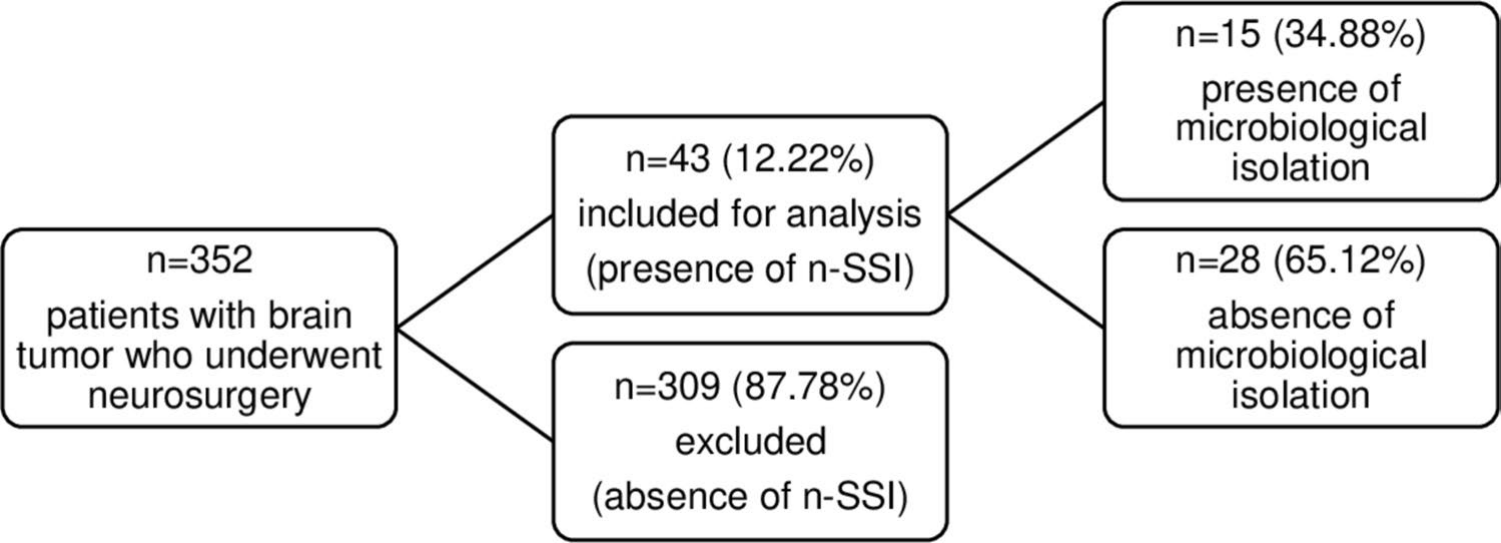
Flow chart of the study population

Of 352 children (0–18 years old) who have undergone neurosurgery for a diagnosis of brain tumor during the study period, 43 (12.22%) children with a suspected/proven n-SSI were considered eligible for this study. Out of these patients only 15 had a confirmed n-SSI, with an infection rate of 4%. The flowchart of the study population is shown in Fig. 1.

**Fig. 2:**
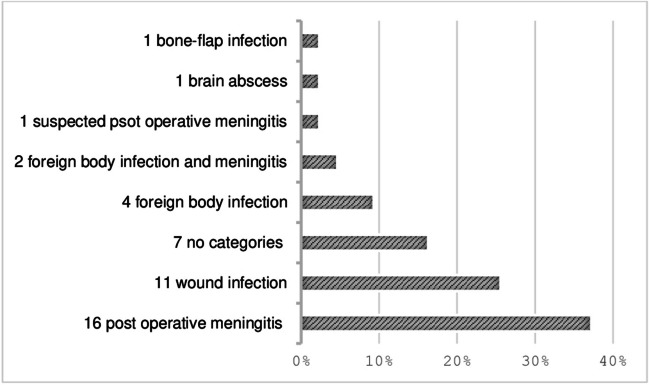
Classification of the type of n-SSIs we found in our study

The classification of the type of n-SSI is reported in Fig. 2. The most frequent one was postoperative meningitis (16/43, 37.2%), followed by superficial/deep wound infection (11/43, 25.6%). Seven patients (16.3%) with suspected n-SSI did not enter in any of the above categories, as they presented with fever and increased inflammatory markers and responded to antibiotic therapy without any microbiological isolation and without signs of localization.

Patients with n-SSI were divided into different subgroups considering the presence or absence of a confirmed microbiological diagnosis, and the presence or absence of an intracranial foreign body. The general characteristics of the study population, according to these subgroups, are summarized in Table 1.

**Table 1:** General characteristics of the study population with suspected/confirmed n-SSI

Variables	All (*n* = 43)	Microbiological isolation (*n* = 15)	Without microbiological isolation (*n* = 28)	*P* value	Foreign body (*n* = 32)	Without foreign body (*n* = 11)	*P* value
Sex, *n* (%)							
F	21 (48.8)	9 (60)	12 (42.9)	0.35	15 (46.9)	6 (54.5)	0.74
M	22 (51.2)	6 (40)	16 (57.1)		17 (53.1)	5 (45.5)	
Age at infection (years)							
Median (IQR)	6 (2.5; 12)	6 (2; 10)	6.5 (3.75; 12)	0.33	6 (3.75; 12)	7 (2; 10.5)	0.78
Type of tumor, *n* (%)							
Low grade	29 (67.4)	12 (80)	17 (60.7)	0.31	20 (62.5)	9 (81.8)	0.29
High grade	14 (32.6)	3 (20)	11 (39.3)		12 (37.5)	2 (18.2)	
Second surgery, *n* (%)							
Yes	23 (53.5)	10 (66.7)	13 (46.4)	0.34	19 (59.4)	4 (36.4)	0.29
No	20 (46.5)	5 (33.3)	15 (53.6)		13 (40.6)	7 (63.6)	
Fever, *n* (%)							
Yes	41 (95.3)	14 (93.3)	27 (96.4)	0.99	31 (96.9)	10 (90.9)	0.45
No	2 (4.7)	1 (6.7)	1 (3.6)		1 (3.1)	1 (9.1)	
CSF leak, *n* (%)							
Yes	23 (53.5)	7 (46.7)	16 (57.1)	0.54	15 (46.9)	8 (72.7)	0.17
No	20 (32.6)	8 (53.3)	12 (42.9)		17 (53.1)	3 (27.3)	
Antibiotic therapy duration median, days (IQR)							
	16 (10; 30.25)	24 (11.25; 34.75)	15 (10; 25.75)	0.26	15 (10; 28.75)	27 (15.5; 34)	0.23
Antibiotic therapy changes							
Yes	28 (65.1)	9 (60)	19 (67.9)	0.74	22 (68.8)	6 (54.5)	0.47
No	15 (34.9)	6 (40)	9 (32.1)		10 (31.2)	5 (45.5)	
Timing of lumbar puncture (*n* = 36)							
Pre-antibiotics	27 (75.0)	13 (100.0)	14 (60.9)	**0.01**	21 (77.8)	6 (66.7)	0.66
Post-antibiotics	9 (25.0)	0 (0.0)	9 (39.1)		6 (22.2)	3 (33.3)	
Delayed chemotherapy (*n* = 20)							
Yes	8 (38.1)	3 (75)	5 (31.3)	0.25	8 (50)	0 (0)	0.12
No	12 (61.9)	1 (25)	11 (68.7)		8 (50)	4 (100)	
Steroids (*n* = 30)							
Long course	10 (33)	2 (20)	8 (50)	0.21	7 (35)	3 (50)	0.64
Short course	16 (53)	8 (80)	8 (50)		13 (65)	3 (50)	

Overall, low-grade tumors were the most frequent type of tumor seen (29/43; 67.4%). However, about half of the patients (21/43, 48.8%) needed adjuvant chemo/radiotherapy after surgery, based on the histology of the lesion. The site of surgery was more frequently the posterior cranial fossa (23/43, 53.5%), followed by suprasellar (13/43, 30.2%) and hemispheric (7/43, 16.3%) areas. All patients underwent antibiotic prophylaxis according to the hospital protocol, with a single dose of cefazolin, repeated if the length of the intervention was more than 6 h, or vancomycin in case of beta-lactamase allergy. We identified 9/43 (21%) patients with previous conditions: however, most of these (7/43, 16%) were endocrinological premorbidities that were likely linked to previous treatments for the brain tumor. More specifically, premorbidities included post-surgical diabetes insipidus, central hypothyroidism, precocious/delayed puberty, post-surgical panhypopituitarism, hypocortisolism post pituitary gland hemorrhage, short stature, and growth deficits. One patient had juvenile monocytic leukemia (JMML) diagnosed at 8 months of age and healed without any treatment; another patient had idiopathic psoriasis. The median number of surgical interventions preceding the infection was 1.4 (IQR 0;1).

In most patients (32/43, 74.4%), an intracranial foreign body was present at the time of infection. Half of them (18/32, 56.3%) had a dural substitute, in particular Gore-Tex DM® (W.L. Gore & Associates Inc., Phoenix, AZ, USA), 8/32 (25%) had a CSF shunt, 5/32 (15.6%) had both, and one patient (3.1%) had a synthetic bone substitute. At the time of infection, most children (30/43, 68%) were taking steroids; particularly, 4/30 (13%) were taking steroid replacement therapy for concomitant central problems (e.g., surgery-related hypopituitarism). The remaining patients were divided into a long-course therapy group (10/30, 33%) and a short-course group (16/30, 53%), with an arbitrary cutoff of 10 days. Among these two groups, the median days of steroid therapy before the infection were 7.5 (IQR 5–10); patients on long-course therapy were less likely to have microbiological positivity compared to short-course therapy (odds ratio 0.25), although such difference was not statistically significant (*p* = 0.21). We also found that 13.9% of patients (6/43) received chemotherapy within 2 months before the infection, with a median time from the last cycle to the infection of 53 days (IQR 46–60). There was no difference in terms of positivity of microbiological testing between those who received chemotherapy within 2 months prior to the infection and those who did not (*p* = 0.65). Most patients showed fever (95.3%, 41/43) and more than half of n-SSI had CSF leak from the surgical wound (53.5%, 23/43). The management of CSF leak differed based on the type of patient, his clinical condition, and the presence of a working shunting device: in 3 cases, hospitalization, CSF culture, and the management of the n-SSI were sufficient to address the CSF leak, once a negative CT scan ruled out the possibility of obstructive hydrocephalus; 30% of patients with CSF leak (7/23) underwent surgical revision and/or needed a temporary external ventricular drainage, and 11/23 patients (48%) had a few stitches applied to the surgical wound and/or had one or more evacuative lumbar punctures performed. In the 2 remaining cases, we could not obtain further information regarding the management of CSF leak.

The median time between the first surgical intervention and the onset of clinical signs of infection was 6 days (IQR 5–11) for CSF leak and 9.5 days (IQR 5–14.25) for fever. No significant association was found between having fever or CSF leak and the presence of an isolation (*p* = 0.99 and *p* = 0.54, respectively) or with having a foreign body placed (*p* = 0.45 and *p* = 0.17, respectively).

More than half of patients (*n* = 23/43, 53.5%) underwent a second surgery after developing infection, with a median time between first and second surgery of 19 days (IQR 15;34). In about two-thirds of subjects (16/23, 69.57%), the foreign body was removed during the second operation due to the suspicion of being the source of infection. However, no significant association was found between having a foreign body placed and a confirmed microbiological diagnosis (*p* = 0.99).

LP was performed in most cases (*n* = 36, 83.7%), but only in 75% of those (27/36) before starting antibiotic therapy. Having an LP performed before the antibiotic start was significantly associated with the finding of a microorganism on the CSF (*p* = 0.01).

In one-third of the patients (15/43, 34.9%), a pathogen was isolated, by culture in 53.3% (8/15) cases, by PCR in 20% (3/15), and by both methods in 26.7% (4/15) cases. The finding of a microbiological isolate was not significantly correlated with the presence of a foreign body (*p* = 0.47). The most common bacteria found was *Staphylococcus aureus* (6/15; 40%), followed by *CoNS* in one-third of the cases (33.3%; 5/15). An antibiotic therapy was started in all patients, with a median duration of 16 days (IQR 10–30.25). The duration of antibiotic therapy was not significantly associated with a confirmed diagnosis of n-SSI (*p* = 0.26), nor with having a FB placed (*p* = 0.23). In 65.1% of cases (28/43), antibiotics were changed by switching or adding drugs. In patients with microbiological isolates, this change was performed in more than half of the patients (9/15, 60%) to target the antibiotic therapy. Moreover, four children needed antibiotic switch due to rash during vancomycin treatment.

## Discussion

To date, this is the first Italian study aiming to describe the epidemiological, clinical, microbiological, and therapeutic characteristics of n-SSI in pediatric patients with brain tumors undergoing neurosurgery. In our population, the rate of suspected or confirmed n-SSI after neurosurgery for brain tumors was 12.22% (43/352). This rate is higher compared to those found in two other reports from European countries analyzing children undergoing neurosurgery for posterior fossa tumors, with a n-SSI rate of 2.04% (1/49) and 3.7% (3/82 cases) respectively [26, 27]. However, both studies only considered confirmed cases of n-SSI, which could explain the lower incidence compared to our population. When only considering n-SSI confirmed by a microbiological diagnosis, the infection rate in our study drops down to 4%, in line with the studies. Other data on n-SSI rate are available from studies involving children who underwent neurosurgical procedures not affected by brain tumor. Children with hydrocephalus undergoing CSF shunt placement had a n-SSI rate of 3.9% (range 0–13%), without significant differences between shunt insertion, shunt revision, or shunt insertion after infection [28]. One paper reported n-SSI rate of 10.3% in patients with an external ventricular drain, including children undergoing surgery for brain tumors, craniosynostosis, and Chiari malformation [29], and another study described a rate of 13.9% positive CSF cultures, and an additional 4% of CSF-pleocytosis with negative cultures, after shunt placements [30], with an overall rate of 18.85%. Conversely, an overall rate of 4.2% of n-SSI was described in a 12-year period study of ventriculoperitoneal shunt insertion in a pediatric center in Singapore [31]. Another paper on 199 children undergoing CSF diversion surgeries described an infection rate of 8.5% but the study does not only consider children affected by brain tumors, limiting the comparison of rates [33]. A confirmed microbiological diagnosis would consent to target antibiotic therapy, but the diagnostic yield of cultures depends on some variables, such as the infection site and the timing of sample collection, particularly related to antibiotic start. In our study, a confirmed microbiological diagnosis was attained in about one-third of cases (15/43, 35%). In another report, the confirmation rate was higher (17/23, 73.9%) [30], while others only described patients with positive cultures; therefore, comparisons were not feasible [32, 33]. Our study shows a significant association between a microbiological isolate and LP before starting the antibiotics was found (*p* = 0.01), confirming the importance of early LP in these patients. This is becoming even more important nowadays, due to the diffusion of multi-drug resistant bacteria in the nosocomial environment, which urges the clinician to obtain a microbiological isolate and its antimicrobial sensitivities, in order to target the antibiotic treatment. Other variables studied in the literature, such as age, gender, histology, foreign body placement, presence of fever, or CSF leak, seems to not influence the yield of microbiological isolate [1–5, 7, 10, 11, 18]. In our study, the most prevalent microorganism isolated was *S. aureus* (40%), followed by coagulase-negative staphylococci (33%). The prevalence of *S. aureus* is higher than in another Italian study conducted in adult patients which only evaluated cases of meningitis after neurosurgery (13%) [9]. Conversely, the prevalence of S. aureus in our study is lower if compared to the rate of n-SSI reported in the American literature (50–75%) [2, 3, 7]. It is important to note that no cases of methicillin-resistant *S. aureus* (MRSA) were found. On the other hand, CoNs etiology was very similar in various studies conducted on both adult and pediatric patients (35% and 25%, respectively), in which the authors used more restrictive criteria for defining n-SSI [1, 8]. Gram-negative bacteria were found in two cases (2/15, 13%), *Acinetobacter baumanii* cultured in a patch and *Haemophilus influentiae* isolated from CSF. According to data from the literature, the prevalence of Gram-negative bacteria in n-SSI varies from 5 to 31%, depending on the population studied and the neurosurgical procedure [1–3, 5]. In the available literature, having an intracranial foreign body placed, particularly a CSF shunt, is a known independent risk factor for developing neurosurgical SSI, even in the pediatric populations [3, 6, 7, 14]. In our population, no significant association was found between fever and placement of a foreign body. Neither of the examined clinical signs was associated with microbiological positivity either. Moreover, among patients with n-SSI, a CSF leakage does not correlate with the presence of a foreign body nor with the acquisition of a microbiological isolate. However, both the presence of fever and CSF should raise the suspicion of infection related to neurosurgery [29]. It is important to note however that our retrospective study analyzed the characteristics of a selected population of suspected/confirmed n-SSI; therefore, no data on risk factors for infection could be extrapolated. Some studies also correlated the incidence of SSI with the number of neurosurgical procedures and the need for a shunt revision [7, 17]. It is interesting to notice that about 60% of patients with a foreign body in our study underwent a second surgery after developing n-SSI versus 36% of patients without a foreign body. Half of the patients with a foreign body had it removed due to the suspicion that it could be the source of infection, pointing out that treatment of n-SSI relies not only on the use of antibiotic therapy, but also on surgical treatment to achieve infection source control. All patients in this study received antibiotic therapy for suspected or confirmed n-SSI but in most cases it was modified during treatment, according to the principles of antimicrobial stewardship. However, neither the duration of therapy nor the need to switch antibiotics were influenced by the presence of a foreign body or by the isolation of an etiologic agent. Lastly, we analyzed the potential delay of adjuvant chemo or radiotherapy in patients with n-SSI and we found that 38% of patients could not start the adjuvant treatment according to the protocol schedules. Particularly, 75% of patients with positive microbiological isolation had to delay chemo or radiotherapy versus 31% of patients without any isolate; however, these data did not reach statistical significance. This study has a retrospective design; therefore, it is limited by the fact that it is based on medical records of patients, with the possibility of lacking precise data, underestimation, and a recall bias. To reduce such bias, we excluded patients with insufficient information. On the other hand, the small sample numerosity due to the paucity of pediatric patients with brain tumors and post-neurosurgery infections does not allow for solid statistical conclusions. Despite these limits, our study highlights the importance of a multidisciplinary evaluation and collaboration between neurosurgeons and pediatric infectious disease specialists when dealing with patients with n-SSI. A large multicenter prospective cohort study would be desirable to better describe characteristics of n-SSI in the pediatric oncologic population, and particularly in some subgroups, such as children with a foreign body, and the variables associated with obtaining a microbiological isolate.

## Conclusion

This is the first Italian report of the epidemiological, clinical, microbiological, and therapeutic aspects of n-SSI in pediatric patients with central nervous system tumors. Our findings reveal a high rate of suspected or confirmed n-SSI (12.22%), underscoring the importance of early and precise diagnostic measures, such as LP prior to antibiotic therapy, to enhance microbiological isolation and target antimicrobial treatment. *Staphylococcus aureus* as the most prevalent pathogen is consistent with literature reports, though the absence of MRSA is noteworthy. The data also suggests that foreign bodies (dural/bone substitute and CSF shunt) do not seem to significantly correlate with clinical signs such as fever or CSF leak, nor with microbiological positivity, highlighting the complex role of factors contributing to infection risk and diagnosis. Surgical intervention plays a critical role alongside antibiotic therapy in managing n-SSI, which remain a key concern for neurooncological patients for its potential of delaying chemotherapy and radiotherapy. Despite the limitations of its retrospective design and small sample size, this study provides valuable insights into the management of n-SSI in pediatric patients with brain tumors, advocating for a multidisciplinary approach. Future prospective studies are recommended to better elucidate the risk factors and outcomes associated with n-SSI to improve clinical decision-making and patient outcomes in this vulnerable population.

## Data Availability

No datasets were generated or analysed during the current study.
